# Laparoscopic left pancreatectomy for pancreatic sarcomatoid carcinoma: A case report and review of the literature

**DOI:** 10.3892/ol.2013.1411

**Published:** 2013-06-18

**Authors:** JIE YAO, JIAN-JUN QIAN, CHANG-REN ZHU, DOU-SHENG BAI, YI MIAO

**Affiliations:** 1 Departments of Hepatobiliary and Pancreatic Surgery, The First Affiliated Hospital of Yangzhou University, Yangzhou, Jiangsu 225001, P.R. China;; 2 Pathology, Northern Jiangsu People’s Hospital, The First Affiliated Hospital of Yangzhou University, Yangzhou, Jiangsu 225001, P.R. China;; 3 Center of Pancreatic Surgery, The First Affiliated Hospital of Nanjing Medical University, Nanjing, Jiangsu 210029, P.R. China

**Keywords:** sarcomatoid carcinoma, pancreas, left pancreatectomy

## Abstract

Sarcomatoid carcinoma of the pancreas is extremely rare. The current report presents a case of carcinosarcoma of the pancreas in a 48-year-old male. Pre-operative computed tomography scans revealed a large complex cystic and solid mass in the tail of the pancreas; the patient underwent a laparoscopic spleen-preserving left pancreatectomy. The tumor was shown to be made of cystic and solid components, with a grossly grey/ white appearance. A histological evaluation of the tumor revealed two elements separated from each other, one component was a pancreatic ductal adenocarcinoma and the other component exhibited a sarcomatous growth pattern, composed of spindle cells and multinucleated giant cells. Immunohistochemically, the epithelial area was positive for cytokeratin (CK) and negative for vimentin, while the sarcomatoid area was negative for CK and positive for vimentin. These observations confirmed a diagnosis of pancreatic carcinosarcoma. Although the patient was treated by gemcitabine following surgery, the outcome was extremely poor and the patient succumbed to sarcomatoid carcinoma three months after the treatment.

## Introduction

Sarcomatoid carcinoma is a rare and extremely aggressive malignant tumor, with a mixture of carcinomatous and sarcomatous elements (1). Sarcomatoid carcinoma occurs in diverse locations throughout the body (2), including the upper respiratory tract, genitourinary tract and upper and lower digestive tracts. Pancreatic carcinosarcoma is also extremely rare and only a few cases have been reported in the literature (3–11). Therefore, this disease remains a diagnostic and therapeutic challenge for surgeons. The current case study presents the first successful use of laparoscopic distal pancreatectomy for a case of pancreatic sarcomatoid carcinoma and discusses the associated literature. Written informed consent was obtained from the patient.

## Case report

### Patient presentation

**Figure 1.**
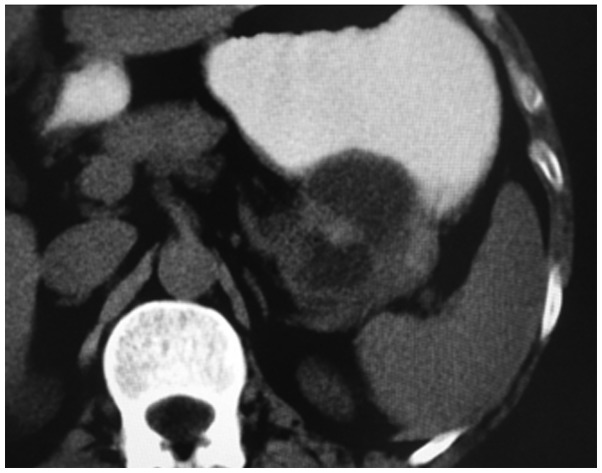
CT scan of the abdomen revealing a large complex cystic and solid mass in the body and tail of the pancreas. CT, computed tomography.

A 48-year-old male presented with a five-month history of epigastralgia, with weight loss of ∼3 kg in one month. One month prior to hospitalization, a gastroscopy revealed an ulcer in the duodenal bulb and chronic superficial gastritis. The patient did not respond to anti-ulcer therapy. There was no remarkable past medical history with no alcohol consumption or history of smoking. In addition, the patient’s family medical history was unremarkable. A physical examination revealed epigastric tenderness upon palpation, however, no palpable abdominal masses were identified. Laboratory examinations reported the following results: Red blood cell (RBC) count, 4.75×10^12^ cells/l; hemoglobin (HB), 144 g/l; white blood cell (WBC) count, 7.3×10^9^ cells/l; albumin (ALB), 43.9 g/l; total bilirubin (TBIL), 19.9 *μ*mol/l; direct bilirubin (DBIL), 2.8 *μ*mol/l; aspartate aminotransferase (AST), 30 U/l; and alanine aminotransferase (ALT), 13 U/l. The carbohydrate antigen (CA)19-9, CA50, α-fetoprotein (AFP) and carcinoembryonic antigen (CEA) levels were 134 U/ml, 50 U/ml, 49 ng/ml and 12 ng/ml, respectively. Abdominal ultrasonography revealed an ∼8×5 cm cystic mass in the tail of the pancreas. Computed tomography revealed a large complex cystic and solid mass in the tail of pancreas (Fig. 1).

### Surgical procedures

**Figure 2.**
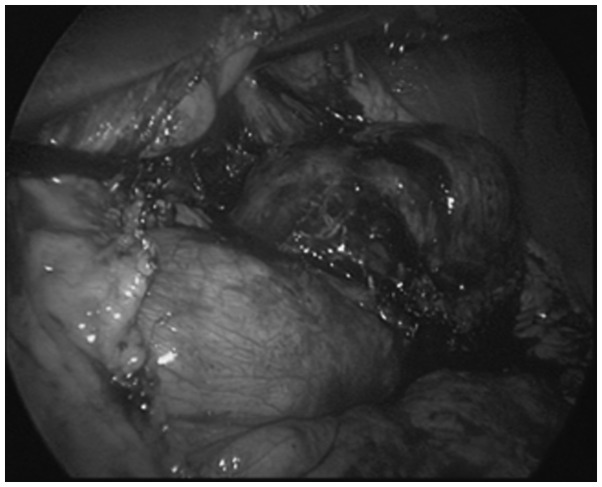
A cystic tumor with intact peplos was detected in the pancreatic tail by exploratory laparoscopy. The patient consequently underwent a laparoscopic spleen-preserving left pancreatectomy.

Exploratory laparoscopy was performed due to a suspected pancreatic cystadenoma. A cystic tumor with intact peplos was detected in the pancreatic tail (Fig. 2). As a consequence, the patient underwent a laparoscopic spleen-preserving left pancreatectomy. There were no post-operative complications, including intra-abdominal bleeding or pancreatic fistula.

### Histopathology

A histopathological analysis revealed that the specimen consisted of a distal portion of the pancreas and the mass, measuring 10×8×5 cm. The surface of the specimen was smooth and sectioning of the pancreatic mass revealed a well-circumscribed tumor with solid and multicystic components. The cut surface of the solid area was grey/white and medium-hard.

### HE staining

A large number of diffuse, abnormal cells were identified by HE staining. Mitotic cells were common and duct-like structures and small cystic formations were identified in specific portions of the tumor. In addition, certain areas of the tumor contained spindle cells with large, pleomorphic nuclei. Multinucleated giant cells were also observed.

### Immunohistochemistry

**Figure 3.**
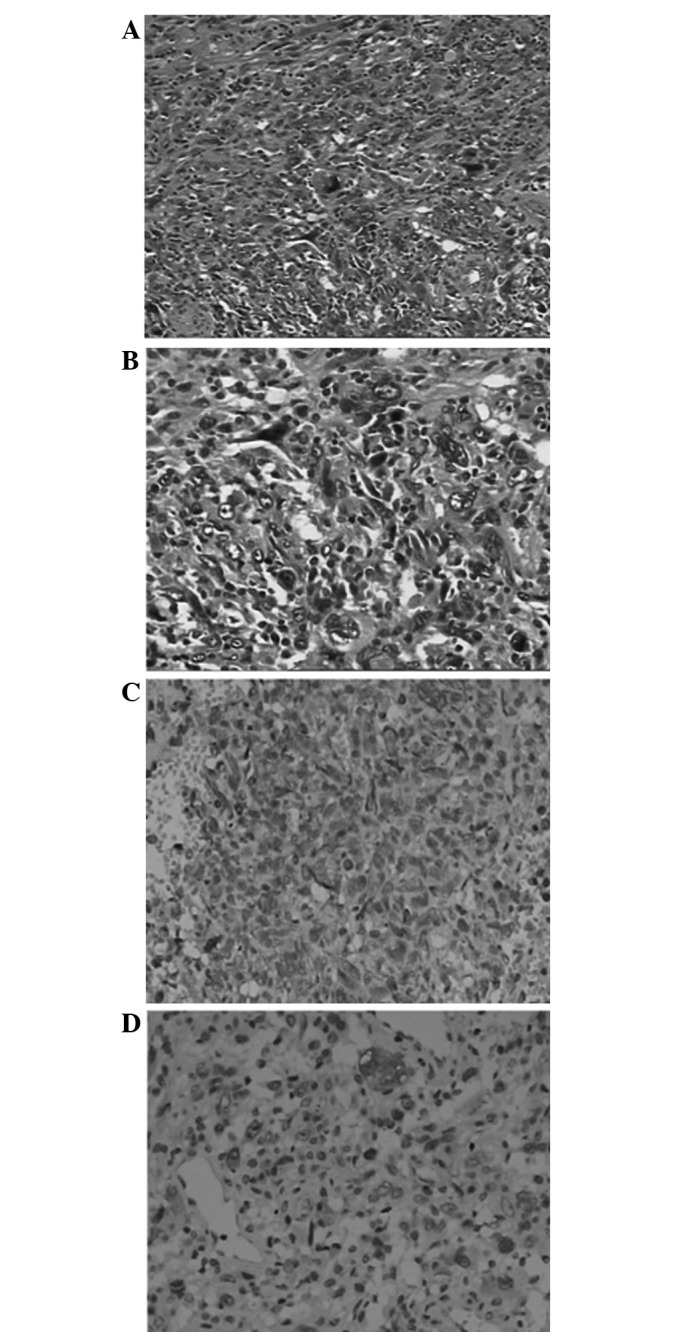
HE staining revealing a tumor composed of (A) diffusely spindle-shaped atypical cells (magnification, ×100) and (B) scattered adenocarcinoma cell clusters (magnification, ×200). Immunohistochemistry identifying that the (C) stromal component exhibited positive vimentin staining (magnification, ×200) and that the (D) adenocarcinoma revealed markedly positive CK18 staining with negative stroma (magnification, ×200).

The adenocarcinoma component was markedly reactive for antibodies against CK18 (++). The sarcomatous component was negative for CK18 antibodies, but markedly reactive to vimentin antibody (++; Fig. 3). Following surgery, the patient received one cycle of chemotherapy with gemcitabine, but succumbed to sarcomatoid carcinoma within three months.

## Discussion

Sarcomatoid carcinoma of the pancreas is a rare neoplasm that contains carcinomatous and sarcomatous components, as demonstrated by immunohistochemical reactivity to cytokeratin and vimentin, respectively. In the present case, the tumor arose in the body of the pancreas. A histological examination revealed a tumor containing solid and multi-cystic components with a large number of abnormal cells, including spindle-shaped and multinucleated giant cells. Immunohistochemistry confirmed mixed adenocarcinoma and sarcomatous components in the tumor. This type of tumor is classified as a sarcomatoid carcinoma, according to the classification by the World Health Organization (1).

The origin of truly mixed malignant carcinosarcoma is unknown. Several theories have been hypothesized to explain the characteristic biphasic appearance of this form of carcinosarcoma, with histogenetic mechanisms involved in the coexistence of carcinomatous and sarcomatous components in the same tumor. These theories are known as the theories of ‘transformation,’ ‘combination’ and ‘collision.’ ‘Transformation’ indicates that a section of the carcinoma transforms into a sarcomatous element, ‘combination’ describes the development of a tumor from a single stem cell that differentiates into epithelial and mesenchymal tissues and ‘collision’ refers to the invasion of carcinomatous and sarcomatous elements into each other. van den Berg *et al* studied pancreatic mucinous cystic neoplasms with sarcomatous stroma and identified that the two components of the carcinosarcomas share a common clonal origin. These observations indicated that the two components of the carcinosarcomas are monoclonal neoplasms derived from a single stem cell (5).

The sarcomatous component of the tumor may vary. Millis *et al* previously reported a case of carcinosarcoma with leiomyosarcoma (4), which was confirmed by immunoreactivity of the spindle cells for antibodies against smooth muscle, vimentin and desmin. Sarcomatous components have also been reported as malignant fibrous histiocytoma (3,6), osteoclastic giant cell tumors (7,12,13) and mucinous cystic neoplasms (14). Yamazaki *et al* (8) reported a primitive fibroblastic or mesenchymal character without a specific differentiation component.

Previous studies have reported an extremely poor prognosis for pancreatic carcinosarcoma. Shen *et al* (9) reported a carcinosarcoma of the pancreas with liver metastasis combined with a gastrointestinal stromal tumor of the stomach in a 72-year-old female. A radical resection, including a pancreaticoduodenectomy, left hepatic lobe resection and local resection, of the gastric mass was performed, however, the patient succumbed to multiple organ failure at 2 months post-surgery. By contrast, in other cases, survival periods of ≤20 months have been reported (10,11). However, 6 months appears to be the most common period for survival (4–7,14).

For pancreatic adenocarcinoma, a laparoscopic pancreatectomy provides similar short- and long-term oncological outcomes to open surgery (15). No difference in surgical duration, margin positivity, incidence of post-operative pancreatic fistula and mortality (16). Due to the small number of cases of carcinosarcoma of the pancreas, a randomized control trial to determine the efficacy of surgery for this condition has yet to be performed. In addition, a standard chemotherapy protocol has not been developed. The diagnosis of pancreatic carcinosarcoma remains controversial. In the present case, prior to surgery, the patient was diagnosed with suspected pancreatic cystadenoma as pancreatic carcinosarcomas exhibit no characteristic symptoms or CT scan results. It is extremely difficult to generate a correct pre-operative diagnosis and therefore, in this case, a laparoscopic exploration and resection was performed. Although, a negative surgical margin was obtained, the tumor reoccurred and the patient succumbed to sarcomatoid carcinoma within 3 months.

In summary, the current case study presents a case of pancreatic carcinosarcoma confirmed by pathological and immunohistochemical analysis. To the best of our knowledge, this is the first report of a laparoscopic left pancreatectomy for pancreatic carcinosarcoma. Although pancreatic carcinosarcoma is rare, it must be considered in the differential diagnosis of a pancreatic tumor. Despite the development of modern diagnostic techniques, the formation of a pre-operative diagnosis remains challenging and if in doubt, an analysis of intraoperative frozen sections may be necessary to select the correct surgical approach.
